# Systematic inflammatory indicators and clinical management of exogenous endophthalmitis due to metal penetrating injury of eyeball

**DOI:** 10.3389/fmed.2024.1466530

**Published:** 2024-12-09

**Authors:** Donghai Wu, Yuan Lin, Huping Wu, Jinhong Cai

**Affiliations:** ^1^Xiamen Eye Center and Eye Institute of Xiamen University, School of Medicine, Xiamen, China; ^2^Xiamen Clinical Research Center for Eye Diseases, Xiamen, China; ^3^Xiamen Key Laboratory of Ophthalmology, Xiamen, China; ^4^Fujian Key Laboratory of Corneal & Ocular Surface Diseases, Xiamen, China; ^5^Xiamen Key Laboratory of Corneal & Ocular Surface Diseases, Xiamen, China; ^6^Translational Medicine Institute of Xiamen Eye Center of Xiamen University, Xiamen, China

**Keywords:** endophthalmitis, ocular trauma, intraocular foreign body, risk factor, inflammatory indicators

## Abstract

**Objective:**

To evaluate systemic inflammatory markers in cases of exogenous endophthalmitis caused by metal foreign bodies after penetrating eye injury and identify risk factors for poor control post-initial emergency surgery.

**Methods:**

Twenty-nine patients with exogenous endophthalmitis underwent emergency surgery with vitrectomy at Xiamen Eye Center (2016–2024). Evaluations included systemic inflammatory markers, microbiology, clinical presentation, treatment strategies, complications, prognostic factors, and visual outcomes. Correlation analysis was performed between blood test results and disease control after initial surgery.

**Results:**

The median patient age was 44.8 ± 16.9 years, with an average treatment delay of 6.1 ± 10.6 days. Males represented 86.2% (*n* = 25), and 41.3% (*n* = 12) had metallic intraocular foreign bodies, which correlated with faster symptom onset (*p* < 0.05) and higher inflammatory markers (WBC, neutrophils, basophils, CRP; *p* < 0.05). Primary lens extraction and intraoperative vancomycin did not significantly improve control (*p* > 0.05). Poor control post-vitrectomy was positively correlated with intraocular foreign bodies (*R* = 0.39, *p* < 0.05) and negatively with lymphocyte and monocyte counts (*R* = −0.43, *p* < 0.05; *R* = −0.46, *p* < 0.05). Early intervention within 2 h of symptom exacerbation reduced complication risk (AUC = 0.708, 95% CI: 0.547–0.838, *p* = 0.047).

**Conclusion:**

Metallic intraocular foreign bodies provoke stronger systemic inflammatory responses, posing control challenges, especially in patients with lower immune resilience. Timely vitrectomy during initial emergency surgery is crucial for managing endophthalmitis.

## Introduction

Endophthalmitis is an infection characterized by inflammatory response in the posterior segment of the eye often triggered by pathogenic microorganisms. It constitutes an ophthalmic emergency due to its potential to cause significant vision loss and complications (1). Following an ocular injury, acute infectious endophthalmitis may develop irrespective of the presence of foreign bodies (2). Intraocular foreign body (IOFB) injury is defined as any object that penetrates the eyeball and remains within the eye, representing a severe form of ocular trauma (3–5). Exogenous endophthalmitis is a serious complication associated with IOFBs, often adversely affecting the visual function of the injured eye and leading to blindness (6).

Exogenous endophthalmitis may occur due to exposure to sharp objects, foreign materials, or occupational hazards. These incidents can lead to the penetration of the eyeball, damaging its delicate structures and facilitating the entry of harmful microorganisms, thereby resulting in infections within the intraocular tissues. The presence of a foreign object lodged in the eye significantly increases the risk of developing infectious endophthalmitis. Therefore, in cases where endophthalmitis is either suspected or confirmed, immediate intervention is essential to prevent progression. Such interventions include the extraction of the intraocular foreign body and the administration of systemic broad-spectrum antibiotics. In cases of advanced severity, vitrectomy may be indicated (7). Vitrectomy is a medical procedure that can help diagnose and treat endophthalmitis. During the procedure, the vitreous is removed and tested to determine if microorganisms are present. Vitrectomy not only removes microorganisms and their toxins but also allows medication to target the affected area directly (8).

This study investigates the systemic inflammatory markers associated with metallic IOFBs in cases of exogenous endophthalmitis following globe-penetrating injuries. Additionally, we report on the clinical outcomes of emergency surgical interventions, which included vitreous vancomycin injection, lens surgery, vitrectomy, and adherence to a standardized clinical protocol. Our analysis aims to elucidate the influence of these factors and treatment modalities on the management of endophthalmitis prior to any subsequent surgical procedures following the initial emergency intervention.

## Method

The research followed the ethical principles outlined in the Declaration of Helsinki. The Human Ethics Committee of Xiamen University, affiliated with Xiamen Eye Center, reviewed and approved the studies involving human participants (XMYKZX-KY-2024-014). All patients provided written informed consent before surgery, including their agreement to use their data in future teaching and research at the institution. We thoroughly analyzed the medical records of every patient, including their emergency records, outpatient records, surgery reports, and any pertinent information.

### Diagnosis, inclusion and exclusion criteria

To determine the presence of endophthalmitis in our research, we defined it as clinical signs of infection and a positive culture growth from intraocular specimens. Clinical indicators of endophthalmitis included a decline in eye function, significant vitreous septic turbidity, purulent discharge within the wound, and retinal vasculitis. Patients who exhibited these symptoms underwent aspiration of diagnostic aqueous and/or vitreous fluid. If the same organism grew on two different media or showed confluent growth on a single medium, it was considered a positive culture of intraocular fluid. Infection was also diagnosed if there was conclusive histological identification of significant bacterial infiltration, even in the absence of a positive culture (9).

The study focused on individuals with specific medical conditions, such as endophthalmitis caused by metal IOFBs. All IOFBs were localized in the vitreous of the affected eyes. Cases involving plant injuries, other foreign objects, and metastatic endophthalmitis were excluded from the analysis.

### Clinical evaluation

The researchers employed a uniform methodology to analyze the patients’ medical records retrospectively, gathering pertinent data such as demographic information, clinical features, underlying infections, and treatment strategies.

Blood tests were initially conducted, including measurements of white blood cell (WBC) count, neutrophils (NEUT), lymphocytes (LYMPH), monocytes (MONO), eosinophils (EOS), basophils (BASO), and C-reactive protein (CRP). Each patient underwent a comprehensive eye examination, which included slit-lamp biomicroscopy. Ultrasonography was performed for cases undergoing primary repair or suspected occult globe rupture. Patients with open ocular injuries deferred their ultrasound evaluation. Computer tomography was utilized for all patients prior to surgical intervention. All treated cases underwent aqueous humor/vitreous puncture, and microbiological tests were meticulously reviewed. Initial and final visual acuity were typically recorded using Snellen acuity and then converted to LogMAR for analysis (10). For counting fingers or worse, the following transformations were used: counting fingers, 2.0 LogMAR; hand movement, 2.3 LogMAR; light perception, 2.6 LogMAR; no light perception, 2.9 LogMAR.

### Treatment strategies

All patients underwent debridement and suturing of ocular penetrating trauma within 24 h of presentation. All initial surgeries were performed collaboratively by two experienced surgeons, Dr. Donghai Wu and Dr. Jinhong Cai, both of whom have over 20 years of clinical experience in emergency ophthalmic trauma. Additionally, all patients received anterior chamber irrigation followed by intravitreal injection of vancomycin antibiotics, combined with vitrectomy. The decision to remove the lens was based on the severity of cataract turbidity. In cases where intraoperative retinal rupture or retinal detachment was observed, endolaser and/or cryoretinopexy was performed on the affected area. Silicone oil was used in all cases of intraoperative retinal detachment (8).

When it comes to treating eye infections, various methods are available, ranging from topical and systemic medications to surgical intervention. For cases with indications or suspicions of bacterial infection, intravitreal injections containing vancomycin at a dosage of 1 mg per 0.1 mL may be prescribed. Post-surgery, systemic antibiotics targeting gram-negative bacteria, such as cephalosporins or gentamicin, are routinely administered intravenously depending on drug allergies. In cases where inflammation is notably severe following vitrectomy, systemic steroids may also be recommended at a dosage of 1 mg per kg of body weight (11).

### Data analysis

We utilized the Fisher exact test or Student *t*-test to compare the demographic data of eyes between two groups, as needed. We applied Kaplan–Meier survival analysis to determine poor control and calculated it between emergency surgery and the second surgery, or between emergency surgery and poor control event. Variables that showed statistical significance in univariate analysis were considered significant if the *p*-value was less than 0.05. Our team performed all data analyses using SPSS 25 version software (IBM, Armonk, New York) and Excel software (Microsoft, Redmond, Washington). We considered *p*-values less than 0.05 significant. The correlation between each indicator was compared, and the correlation heat map was drawn using ChiPlot^1^ (accessed on 18 July 2024).

## Result

This study included 29 patients with exogenous endophthalmitis, with a mean age of 44.8 ± 16.9 years (range: 3–68 years). The average time to treatment was 6.1 ± 10.6 days, with 25 males (86.2%) and four females (13.8%). Patients were grouped based on the presence of metallic IOFB, with demographic and baseline characteristics presented in Table 1. The metallic IOFB group showed significantly elevated systemic inflammatory markers, including leukocytes, neutrophils, basophils, and C-reactive protein (*p* < 0.05; Table 2).

**Table 1:** Demographic, clinical, and treatment information of patients.

	Non-IOFB group	IOFB group	*p*-value
Sex			0.517^b^
Male, *n* (%)	14 (83%)	11 (91%)	
Female, *n* (%)	3 (17%)	1 (9%)	
Age, years	41.76 ± 20.05	49.08 ± 10.30	0.044^a^
Symptom duration, days	8.60 ± 13.26	2.47 ± 2.69	0.006^a^
Before treatment BCVA, LogMAR	2.27 ± 0.49	2.25 ± 0.55	0.765^a^
After treatment BCVA, LogMAR	1.86 ± 0.68	1.53 ± 0.83	0.116^a^
Maximum through-opening diameter, mm	2.53 ± 3.03	3.83 ± 1.80	0.052^a^
Anterior chamber flare, score	1.12 ± 1.36	1.08 ± 1.31	0.892^a^
Corneal penetration			0.769^b^
Yes	15	11	
No	2	1	
Sclera penetration			0.658^b^
Yes	13	10	
No	4	2	
Hypopyon			0.139^b^
Yes	8	9	
No	9	3	
Pupil exudation			
Yes	8	3	0.236^b^
No	9	9	
Presence of lens			0.131^b^
Yes	14	12	
No	3	0	
Combined lens aspiration surgery			0.175^b^
Yes	12	11	
No	5	1	
Intraoperative use of vancomycin			0.100^b^
Yes	11	11	
No	6	1	

IOFB, intraocular foreign body; BCVA, best corrected visual acuity.

a *t-test.*

b *χ2 test.*

**Table 2:** Blood immune-inflammatory indicators of patients (*t*-test).

	Non-IOFB group	IOFB group	*p*-value
WBC, 10^9^/L	10.79 ± 3.26	12.53 ± 5.47	0.011*
NEUT, 10^9^/L	8.06 ± 3.00	9.44 ± 4.79	0.044*
LYMPH, 10^9^/L	2.06 ± 0.85	2.18 ± 1.37	0.168
MONO, 10^9^/L	0.52 ± 0.28	0.72 ± 0.37	0.410
EOS, 10^9^/L	0.06 ± 0.08	0.08 ± 0.09	0.831
BASO, 10^9^/L	0.06 ± 0.04	0.08 ± 0.08	0.035*
CRP, mg/L	1.78 ± 2.67	5.59 ± 9.54	0.046*

IOFB, intraocular foreign body; WBC, white blood cell; NEUT, neutrophil; LYMPH, lymphocyte; MONO, monocyte; EOS, eosinophil; BASO, basophil; CRP, C-reactive protein. ^*^p < 0.05.

**Figure 1:**
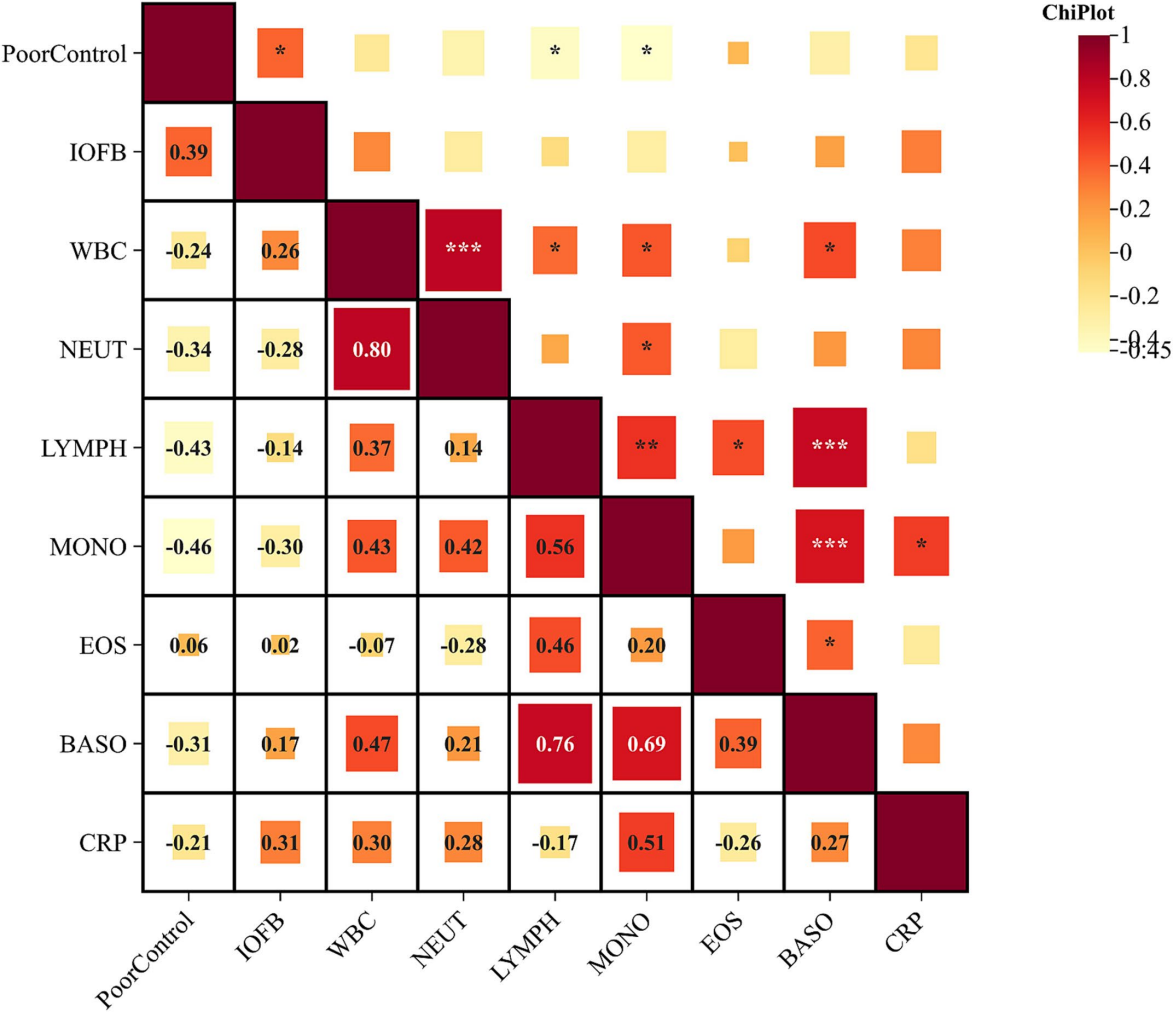
Correlation analysis of systemic blood test and intraocular foreign body and poor postoperative control. Routine systemic blood tests performed before treatment showed that poor postoperative control was associated with lower immune cell levels. * < 0.05, ** < 0.01, and *** < 0.001.

Following initial emergency surgery, five patients experienced uncontrolled inflammation, requiring repeat vitreous injection or vitrectomy due to increasing purulent exudation in the anterior chamber or vitreous. Correlation analysis indicated associations among systemic inflammatory indicators (Figure 1). Poor control after vitrectomy correlated positively with the presence of IOFBs (*R* = 0.39, *p* < 0.05) and negatively with lymphocyte and monocyte counts (*R* = −0.43, *p* < 0.05; *R* = −0.46, *p* < 0.05) (Table 3). Early intervention within 2 h of symptom exacerbation was associated with reduced complications (AUC = 0.708, 95% CI: 0.547–0.838, *p* = 0.047). K–M survival analysis using the log-rank method found no significant difference in outcomes related to primary lens extraction (*p* = 0.203) or the addition of vancomycin infusion (*p* = 0.780).

**Table 3:** Correlation test between poor control and blood tests.

	Poor control	
	*R*	*p*
IOFB	0.39	*p* = 0.038*
WBC	−0.24	*p* = 0.073
NEUT	−0.34	*p* = 0.316
LYMPH	−0.43	*p* = 0.007*
MONO	−0.46	*p* = 0.037*
EOS	0.06	*p* = 0.804
BASO	−0.31	*p* = 0.068
CRP	−0.21	*p* = 0.843

IOFB, intraocular foreign body; WBC, white blood cell; NEUT, neutrophil; LYMPH, lymphocyte; MONO, monocyte; EOS, eosinophil; BASO, basophil; CRP, C-reactive protein. ^*^p < 0.05.

## Discussion

The advancements in ophthalmic microsurgery have e significantly improved the management of endophthalmitis. However, the prognosis for this condition remains suboptimal. Early symptoms and systemic effects from extensive injuries often complicate the timely diagnosis and management of endophthalmitis (12). Acute endophthalmitis triggers an intense inflammatory response, which can result in profound vision loss or, in severe cases, the loss of the eye (13). Treatment outcomes still fall short of achieving consistently favorable prognoses. Early manifestations and systemic responses from widespread trauma can obscure the diagnosis and delay treatment initiation (14). Several factors influence the prognosis of open-globe injuries, including the foreign object’s size and type, retinal or other structural damage, preoperative visual acuity, and surgical duration; these considerations are crucial when planning treatment with patients (15).

Cytokines released from immune cells exacerbate inflammation and fibrosis, with the response varying across among different eye regions in cases of traumatic or pathological injury (16). In this study, we evaluated the influence of metallic IOFB on systemic inflammation and the effects of initial emergency treatment strategies on postoperative outcomes. Our findings suggest that the presence of metallic IOFB can induce a robust inflammatory response, as demonstrated by elevated systemic inflammatory markers. Additionally, patients with lower preoperative leukocyte, lymphocyte, and monocyte counts exhibited an increased risk of poor postoperative control, highlighting the need for heightened surveillance in these cases. This underscores the potential for delayed microbial infection or abnormal immune responses despite apparent stability in ocular signs (17). Systemic broad-spectrum antibiotic therapy remains a common approach for preventing endophthalmitis, with studies indicating that oral antibiotics can achieve clinical outcomes comparable to intravenous antibiotics (18). However, the limited efficacy of antibiotics alone, without adjunctive strategies, may sometimes contribute to persistent disease.

Delayed wound healing, lens capsule rupture, and contamination are risk factors for suppurative endophthalmitis following open-globe injuries (19). Postoperative wound leakage remains a significant complication. Timely and appropriate wound management can mitigate the risks of leakage and subsequent endophthalmitis (20). Patients with IOFB, traumatic cataracts, retinal detachment, extensive wounds, microbial-positive cultures, and work-related injuries typically experience poorer prognoses (21). Shorter wound length, absence of retinal detachment, and better initial visual acuity correlate with improved visual outcomes in open-globe trauma cases (22). Employing corneal laceration suture with intravitreal vancomycin and ceftazidime injection is effective across various healthcare settings (23). Complete removal of the capsule and lens is also deemed beneficial in managing endophthalmitis secondary to open-globe trauma (24). During the initial emergency surgery, 23 cases of cataract resection were performed. However, there was no statistical difference in the postoperative condition of the patients.

Early intravitreal injection of broad-spectrum antibiotics remains an effective treatment for acute endophthalmitis (25). Nonetheless, increasing antibiotic resistance suggests that empiric antimicrobial therapy alone may be insufficient (26). Additional intravitreal antibiotics should be administered with caution, with a 48-h interval recommended if no clinical improvement is noted, in which case early vitrectomy is advisable (27). Vitrectomy following ocular trauma is not only a means of anatomical reconstruction but also plays a role in restoring functional vision. Studies have shown that favorable final visual outcomes are associated with the absence of an afferent pupillary defect, ocular trauma score, preoperative visual acuity, and the location of the injury (28). Therapeutic vitrectomy effectively restores intraocular pressure balance and maintains intraocular stability, leading to improved functional and anatomical outcomes. By promptly reducing the microbial load, vitrectomy enhances the efficacy of intravitreal and systemic antibiotic therapy, creating an optimal recovery environment (29–31). For post-injection endophthalmitis, early vitrectomy combined with subsequent intravitreal antibiotic administration is a practical approach to promote visual recovery (32). The findings of this study indicate that integrating vitrectomy into the initial treatment protocol for metal-induced endophthalmitis may effectively manage inflammation and infection, creating a conducive environment for potential secondary surgical interventions. Moreover, early intervention—specifically within 2 h of symptom exacerbation—was linked to a reduction in complications.

Ruptured open globe injuries and the presence of endophthalmitis are recognized as major risk factors for enucleation, particularly when endophthalmitis presents with no light perception, panophthalmitis, or arises from exogenous sources, which significantly increase the likelihood of evisceration or enucleation (33, 34). At discharge or follow-up, endophthalmitis patients requiring enucleation or evisceration represent a notably higher proportion compared to those without endophthalmitis, reflecting the severity of their intraocular conditions (14). Patients who undergo these procedures typically present with additional complications such as trauma, corneal ulcers, or other endophthalmitis-related causes, and are less likely to receive globe-salvaging surgeries (35). Research suggests that prompt primary repair within 24 h of injury, intraocular tissue prolapse, and spontaneous wound closure may offer some protective benefit against endophthalmitis development (36). Effective and timely management of endophthalmitis remains essential, with immediate vitrectomy and intravitreal antibiotic injection emerging as effective options, especially in cases of post-injection endophthalmitis. Furthermore, early vitrectomy, ideally within the first 24 h, has been associated with improved visual outcomes, underscoring the importance of rapid intervention in these cases (32, 37).

One of the main limitations of this study is its retrospective nature, which may lead to biases in the selection of cases that receive different treatment modalities. Additionally, the data was collected from a tertiary referral center, which means that the volume of data was limited, and some crucial variables may not be reflected. Therefore, the study may not represent data found in other settings.

## Conclusion

This study highlights the critical importance of early intervention in exogenous endophthalmitis, especially with metallic IOFBs, where elevated inflammatory markers correlate with poorer outcomes. Vitrectomy within 2 h of endophthalmitis symptom onset is crucial for controlling infection and inflammation, setting favorable conditions for secondary surgeries if needed.

## Data Availability

The data are not publicly available due to restrictions that apply to the availability of the data (e.g., privacy or ethical). Datasets from this study may be available upon request from the corresponding author and provided upon approval from the sponsor and in accordance with data privacy and ethical provisions. Requests to access these datasets should be directed to YL, yuanlin_huaxiaeye@foxmail.com.

## Footnotes

^1^ https://www.chiplot.online/
